# Direct-fed microbials for enteric methane mitigation in ruminants: a function-first framework for strain selection and farm-scale translation

**DOI:** 10.1093/tas/txag170

**Published:** 2026-10-24

**Authors:** Md Al-Amin Sarker, Rayhan Ahmmed Pranto, Debasree Saha, Moin Uddin, Khatun-A-Jannat Esha, Sabrina Zaman Seema, Kazi Md Al-Noman, Mueena Jahan, Md Morshedur Rahman

**Affiliations:** Department of Dairy and Poultry Science, Faculty of Veterinary Medicine and Animal Science, Gazipur Agricultural University, Gazipur 1706, Bangladesh; Department of Dairy and Poultry Science, Faculty of Veterinary Medicine and Animal Science, Gazipur Agricultural University, Gazipur 1706, Bangladesh; Department of Dairy and Poultry Science, Faculty of Veterinary Medicine and Animal Science, Gazipur Agricultural University, Gazipur 1706, Bangladesh; Department of Dairy and Poultry Science, Faculty of Veterinary Medicine and Animal Science, Gazipur Agricultural University, Gazipur 1706, Bangladesh; Department of Dairy and Poultry Science, Faculty of Veterinary Medicine and Animal Science, Gazipur Agricultural University, Gazipur 1706, Bangladesh; Department of Dairy and Poultry Science, Faculty of Veterinary Medicine and Animal Science, Gazipur Agricultural University, Gazipur 1706, Bangladesh; Department of Dairy and Poultry Science, Faculty of Veterinary Medicine and Animal Science, Gazipur Agricultural University, Gazipur 1706, Bangladesh; Department of Microbiology and Public Health, Faculty of Veterinary Medicine and Animal Science, Gazipur Agricultural University, Gazipur 1706, Bangladesh; Department of Dairy and Poultry Science, Faculty of Veterinary Medicine and Animal Science, Gazipur Agricultural University, Gazipur 1706, Bangladesh

**Keywords:** direct-fed microbials, enteric methane, hydrogen metabolism, methanogenic archaea, rumen fermentation, ruminants

## Abstract

Enteric methane (CH_4_) is both an energy loss from ruminants and an important emission from livestock production. Direct-fed microbials (DFMs) can modify rumen fermentation, but their effects on methane are inconsistent. This narrative review evaluates administered microbial interventions rather than naturally occurring taxa associated with low-methane phenotypes. A focused literature update prioritized in vivo studies that directly measured methane production, yield, or intensity. Animal evidence remains mixed. *Propionibacterium*-based DFMs, lactic acid bacteria, and conventional *Saccharomyces cerevisiae* products often show no change in daily methane, although strain- and diet-specific responses occur. *Bacillus* studies report both modest reductions and null responses. Recent approaches are more encouraging: a methanotroph-methylotroph consortium reduced daily methane by 12.0% to 14.4% in Hanwoo steers, and a rumen-derived *Pichia membranifaciens* strain reduced methane production, yield, and intensity in dairy cows at the higher tested dose. These findings are promising but still require independent and longer-term confirmation. Mechanistically, DFMs should not be described as eliminating ruminal H_2_ production. A more defensible target is redirection of reducing equivalents and H_2_/formate flux toward competing pathways, together with rumen stabilization or specific microbial antagonism. We therefore propose a function-first, evidence-tiered framework for DFM development. Farm-scale claims should require persistent changes in methane production, yield, or intensity without loss of intake, digestibility, health, or productivity.

## Teaser

This review reframes direct-fed microbials as mechanism-dependent, diet-responsive rumen modifiers rather than universal methane suppressors. It proposes a practical framework for strain selection, methane measurement, and farm-scale translation.

## Introduction

Ruminants convert fibrous feeds into milk and meat, but rumen fermentation also produces methane (CH_4_). Methanogenic archaea use reducing equivalents released during anaerobic fermentation, mainly through H_2_- and formate-dependent pathways. Enteric CH_4_ commonly represents about 2% to 12% of gross energy intake, with the proportion strongly influenced by diet and intake (Johnson and Johnson 1995; Janssen 2010). Reducing CH_4_ can therefore contribute to lower emissions from ruminant production while potentially retaining more feed energy for productive processes (Martin et al. 2010; Hristov et al. 2013).

Direct-fed microbials are attractive because microbial products are already used to support rumen function and animal performance. Their methane-mitigation evidence, however, is heterogeneous. A recent cattle meta-analysis found no significant overall effect of probiotics on CH_4_ production, yield, or intensity, although multistrain bacterial products and longer supplementation periods showed more favorable subgroup responses (Ncho et al. 2024). Primary animal studies likewise range from reductions in selected strain-diet combinations to clear null responses (Vyas et al. 2014a, 2014b; Jeyanathan et al. 2016, 2019; Williams et al. 2023).

Two distinctions are important when this evidence is interpreted. First, a resident rumen taxon associated with a low-methane phenotype is not automatically a deployable DFM. Second, a change in fermentation or microbial abundance is not itself proof of methane mitigation. Earlier reviews established the biological rationale for DFMs and highlighted strain-specific responses, but the expanding animal literature now allows a more explicit comparison of efficacy, mechanism, and translational readiness (Jeyanathan et al. 2014; Doyle et al. 2019; Ncho et al. 2024). Recent in vivo studies of *Bacillus*, lactobacilli, native rumen consortia, methanotroph-based consortia, and rumen-derived yeasts further emphasize that microbial effects depend on strain, diet, dose, persistence, and the methane metric used (Jia et al. 2022; Juckem et al. 2025; Tseten et al. 2025; Li et al. 2026; Nair et al. 2026).

This review therefore has four aims. It defines the ruminal hydrogen and methanogenic ecology relevant to microbial interventions; synthesizes in vivo evidence for administered DFMs; develops a function-first framework that separates mechanistic plausibility from controlled and translational efficacy; and identifies the measurements needed before a microbial methane claim is considered farm-ready.

### Literature search and evidence selection

A focused literature update was completed on 6 September 2026. PubMed was used as the primary bibliographic database, supplemented by targeted publisher and DOI searches and backward citation checking of major reviews and the recent cattle meta-analysis. Search terms combined animal descriptors (ruminant, cattle, dairy cow, beef cattle, sheep, and goat) with methane-related terms (methane, CH_4_, and methanogenesis) and microbial-intervention terms (direct-fed microbial, probiotic, Lactobacillus, Lacticaseibacillus, Propionibacterium, Bacillus, Saccharomyces, Pichia, methanotroph, acetogen, and microbial consortium). Earlier studies were retained when they provided foundational information on ruminal methanogenesis, hydrogen metabolism, microbial ecology, or methane-measurement principles.

For evaluation of in vivo efficacy, studies were prioritized when a viable microbial strain, defined microbial mixture, or microbial consortium was administered to live ruminants and enteric CH_4_ was measured directly as daily production, yield relative to feed intake, or intensity relative to animal product. In vitro and RUSITEC studies were used to interpret mechanisms and screen biological plausibility but were not considered evidence of animal efficacy. Studies reporting only associations between naturally occurring rumen taxa and methane phenotype were used as ecological or mechanistic background rather than as DFM efficacy evidence. Non-ruminant studies and reports without methane-related outcomes were excluded from the DFM efficacy synthesis. Non-microbial interventions were considered only where relevant to comparison or combination strategies.

Evidence was mapped rather than statistically pooled because the literature differed substantially in microbial strain, dose, animal species, basal diet, experimental duration, methane-measurement method, and outcome metric. Studies were therefore interpreted according to DFM class, proposed function, animal and diet context, methane outcome, and level of translational readiness. Representative in vivo studies with direct methane measurements are summarized in Table 2, while mechanistic and contextual evidence is integrated throughout the narrative. Figure 1 summarizes the literature-identification and evidence-classification approach.

**Figure 1:**
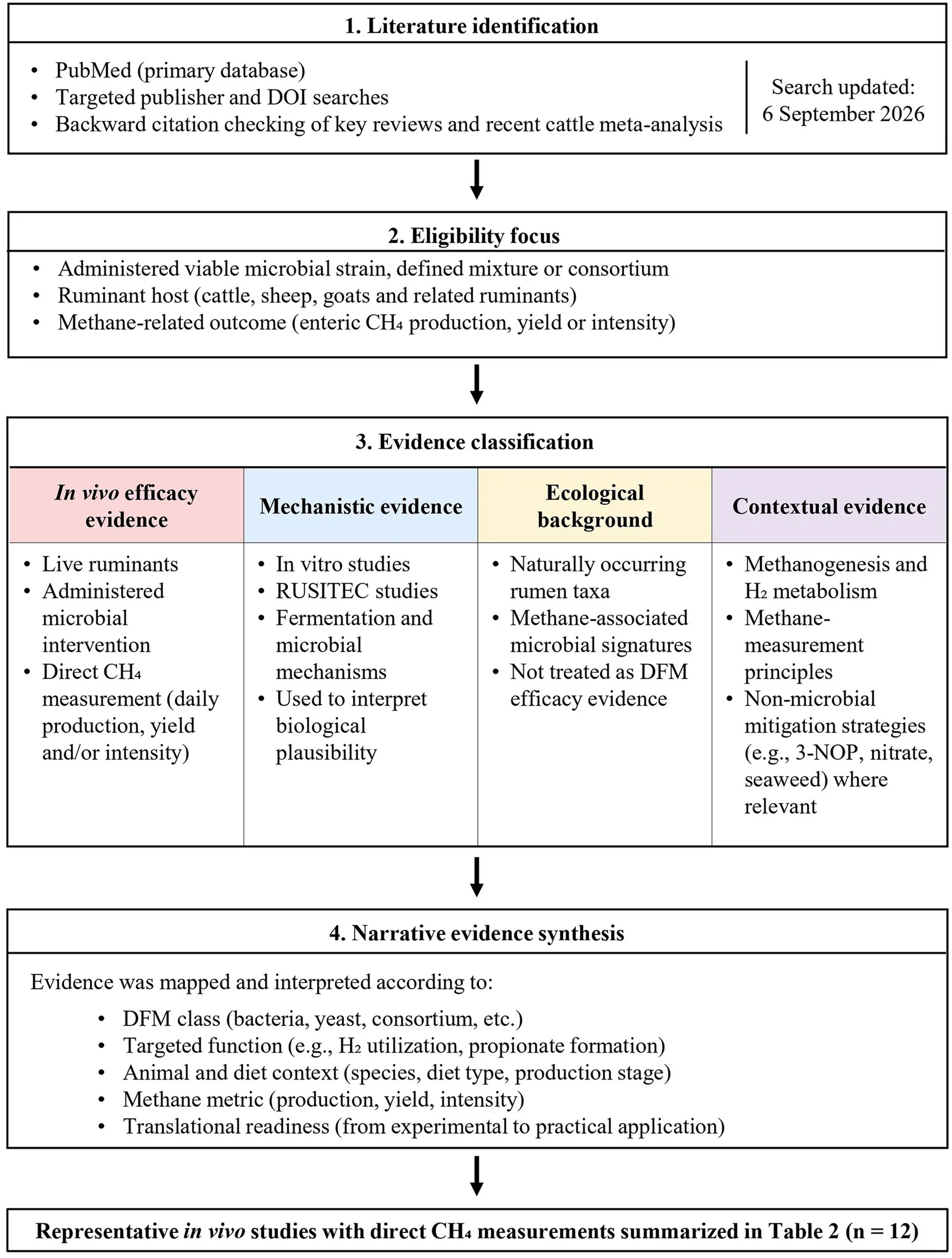
Literature identification and evidence-classification approach used in this narrative review. A focused literature update was conducted using PubMed, targeted publisher and DOI searches, and backward citation checking. Evidence was classified as in vivo efficacy, mechanistic, ecological-background, or contextual evidence and mapped according to DFM class, targeted function, animal and diet context, methane metric, and translational readiness. Twelve representative in vivo studies with direct methane measurements are summarized in Table 2.

**Table 1:** Major ruminal methanogenic archaeal groups relevant to dietary microbial methane mitigation.

Group	Main substrates	Ecological relevance	Implication for DFM strategies
***Methanobrevibacter***	H_2_ + CO_2_ or formate	Often dominant in cattle and sheep; central to hydrogenotrophic methanogenesis.	DFMs are more likely to alter substrate flow or surrounding ecology than to replace this genus directly.
***Methanosphaera***	Methanol + H_2_	Methylotrophic archaeon; abundance varies with host and diet.	Methyl-group substrates should be considered in addition to CO_2_-reducing pathways.
***Methanomicrobium***	H_2_ + CO_2_ or formate	Usually less abundant than *Methanobrevibacter* but part of the hydrogenotrophic network.	Community shifts may occur without a large change in total CH_4_.
***Methanomassiliicoccaceae***	Methylamines or methanol + H_2_	Methylotrophic archaeal lineage often present at lower abundance.	Shows that ruminal methanogenesis involves more than one archaeal genus or substrate route.

DFM, direct-fed microbial; H_2_, molecular hydrogen; CO_2_, carbon dioxide. Ecological summaries are based mainly on Janssen and Kirs (2008), Morgavi et al. (2010), Henderson et al. (2015), Tapio et al. (2017) and Greening et al. (2019). These archaea are biological context, not administered DFMs.

**Table 2:** Representative in vivo evidence for administered direct-fed microbials and related microbial strategies affecting enteric methane in ruminants.

Study	Animal and diet	Microbial intervention	Duration/CH_4_ method	Methane response	Interpretation
**Vyas et al. 2014a**	20 beef heifers; 70:30 forage: concentrate	Three *Propionibacterium* strains; 5 × 10^9^ CFU/d	28-d periods; respiration chambers	Daily CH_4_ unchanged; CH_4_/DMI 8%–13% lower	Intensity response was linked partly to intake; poor strain persistence.
**Vyas et al. 2014b**	20 beef heifers; 10:90 forage: concentrate	Three *Propionibacterium* strains; 5 × 10^9^ CFU/d	28-d periods; enteric CH_4_ measured	CH_4_ production and yield unchanged	High-starch background and existing propionate production may limit response.
**Jeyanathan et al. 2016**	12 rumen-cannulated sheep	Three selected bacterial DFMs	4-wk; repeated in vivo CH_4_	One strain ≈13% lower; one no effect; one ≈16% higher	Strong strain-specificity despite common in vitro screening.
**Jeyanathan et al. 2019**	8 primiparous dairy cows; high- vs low-starch diets	Three bacterial DFMs selected from prior screening	4-wk periods; respiration chambers	No DFM reduced CH_4_; one treatment increased CH_4_ intensity	Clear in vitro-to-in vivo translation failure.
**Oh et al. 2019**	18 lactating Holsteins; 60:40 forage: concentrate	*S. cerevisiae*-based DFM; 28 g/d	28-d periods; enteric CH_4_ measured	Production, yield, and intensity unchanged	Milk yield increased ≈2 kg/d; production benefit ≠ methane suppression.
**Deng et al. 2018**	24 Dorper-cross wethers	*B. licheniformis*; 2.5 × 10^8^ to 2.5 × 10^1^^0^ CFU/d	In vivo methane measurement	Low/mid doses lowered daily CH_4_ and CH_4_/DMI by ≈6%	Modest but biologically coherent response with improved digestibility.
**Jia et al. 2022**	60 lactating Holsteins	*B. subtilis*; 50 g/cow per d	75 d; GreenFeed	CH_4_ yield -10%; intensity -6.8%	Positive response accompanied by greater DMI and milk yield.
**Williams et al. 2023**	40 lactating Holstein-Friesians; vetch hay + grain	Commercial *Lactobacillus* mixture	Controlled feeding; SF_6_	Yield and intensity unchanged	Numerically favorable but not significant.
**Tseten et al. 2025**	12 Hanwoo steers; 70.8% concentrate	Methanotroph-methylotroph consortium NC52PC	Three 30-d periods; enteric CH_4_ measured	Daily CH_4_ -12.0% and -14.4% at low/high dose	Promising emerging consortium; animal sample small and replication needed.
**Juckem et al. 2025**	64 lactating Holsteins; 21% vs 27% starch	*B. subtilis* 810 + *B. licheniformis* 809	12 wk; enteric CH_4_ measured	DFM did not reduce CH_4_	Dietary starch, not DFM, drove CH_4_ reduction.
**Nair et al. 2026**	40 early-lactation grazing Holsteins	Freeze-dried or liquid *Lactobacillus* spp.	35 d; modified SF_6_	No significant effect; freeze-dried group ≈6% lower yield numerically	Pasture context, dose, passage, and colonization may limit effect.
**Li et al. 2026**	36 dairy cows	Rumen-derived *P. membranifaciens* M12; 2.5 or 5 × 10^1^^1^ CFU/d	Randomized block trial; enteric CH_4_ measured	High dose: daily -15.4%, yield -18.7%, intensity -17.7%	Strong recent signal without reduced lactation performance; independent replication needed.

CFU, colony-forming units; CH_4_, methane; DFM, direct-fed microbial; DMI, dry matter intake; SF_6_, sulfur hexafluoride. Percentages are reported study-specific responses and should not be pooled or treated as class-wide effects.

### Ruminal methanogenesis and hydrogen metabolism

Carbohydrate fermentation in the rumen generates volatile fatty acids together with CO_2_, H_2_, formate, and other reduced intermediates. H_2_ concentration is dynamically linked to fermentation thermodynamics. Methanogenic archaea help maintain a low H_2_ partial pressure by consuming H_2_ or formate and reducing CO_2_ or methyl-group substrates to CH_4_ (Janssen 2010; Greening et al. 2019; Ungerfeld 2020).

The main ruminal methanogens are archaea rather than bacteria. Methanobrevibacter commonly dominates hydrogenotrophic methanogenesis in cattle and sheep (Janssen and Kirs 2008; Henderson et al. 2015). Methanosphaera and members of the Methanomassiliicoccaceae are methylotrophic archaea that use methyl compounds with H_2_, whereas Methanomicrobium contributes to the hydrogenotrophic network at lower abundance in many animals (Janssen and Kirs 2008; Tapio et al. 2017). Table 1 summarizes the ecological relevance of these groups without presenting them as DFMs.

Reductive acetogenesis is a potential alternative sink for CO_2_ and H_2_, but it is constrained in the adult rumen. Hydrogenotrophic methanogens generally operate more effectively at the low H_2_ partial pressures typical of this ecosystem, which limits the ability of introduced acetogens to compete consistently (Janssen 2010; van Lingen et al. 2016; Ungerfeld 2020). Accordingly, the realistic DFM target is not to stop normal H_2_ formation. It is to redirect a greater share of reducing equivalents and H_2_/formate flux toward competing H_2_-consuming or reduced-product pathways while maintaining fermentation efficiency (Greening et al. 2019; Ungerfeld 2020). Figure 2 illustrates these intervention points.

**Figure 2:**
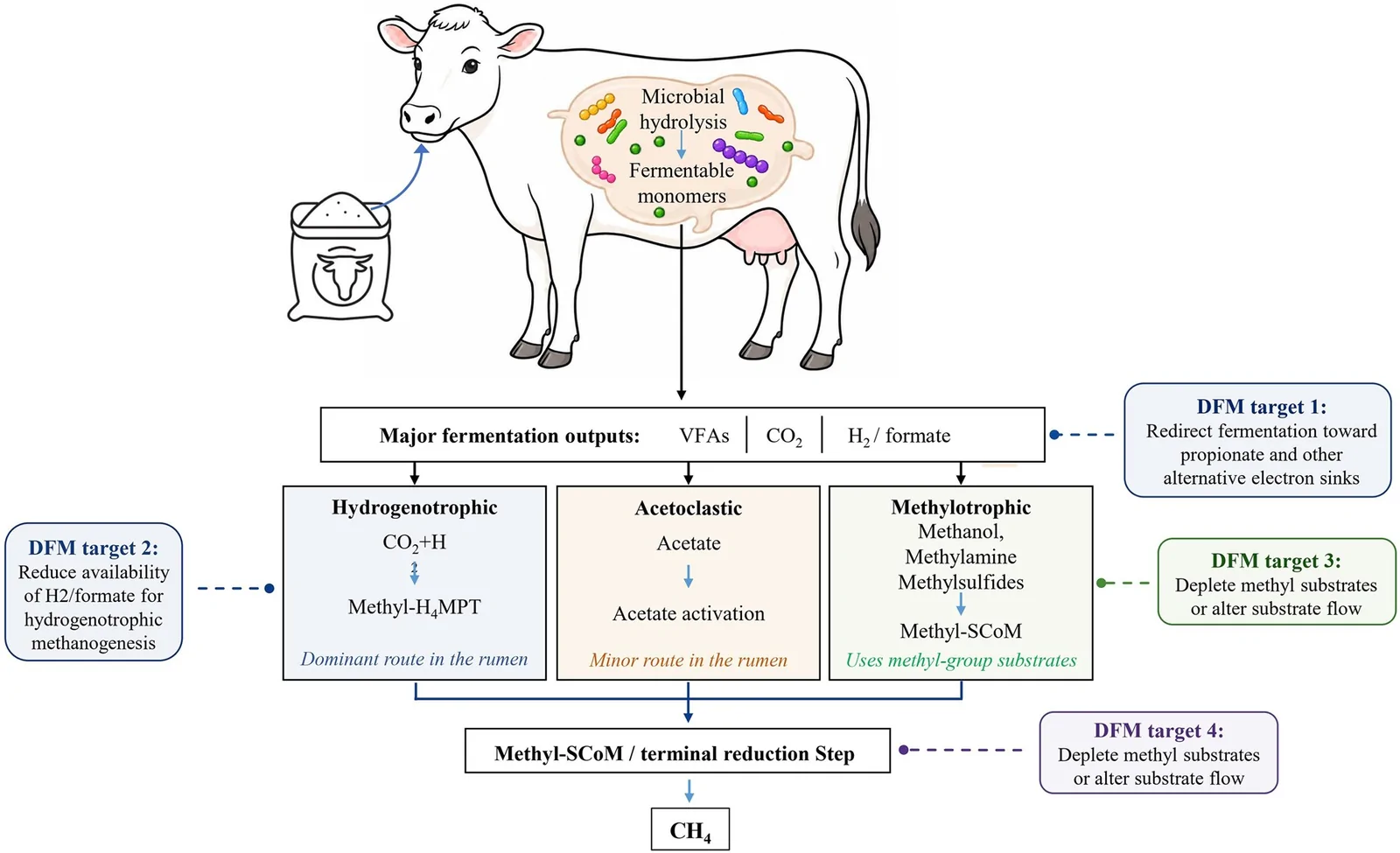
Ruminal methanogenesis and potential DFM intervention points. Anaerobic fermentation generates volatile fatty acids, CO_2_, H_2_, and formate. Hydrogenotrophic methanogenesis is the dominant methane-forming route in the rumen, while methylotrophic archaea use methyl-group substrates and acetoclastic methanogenesis is generally minor. DFM strategies may redirect reducing-equivalent and H_2_/formate flux toward competing pathways, alter methyl-substrate flow, stabilize fermentation, or affect methanogenic activity (Janssen 2010; Greening et al. 2019; Ungerfeld 2020).

### In vivo evidence for DFM effects on enteric methane

The most important limitation of the DFM literature is the small and inconsistent animal evidence base. Ncho et al. (2024) identified 20 cattle articles representing 49 comparisons for meta-analysis. The pooled effects were not significant for CH_4_ production, yield, or intensity. Multistrain bacterial formulations performed better in subgroup analysis, and longer supplementation was associated with larger effects. These results support an evidence hierarchy in which individual animal studies are interpreted by strain, diet, duration, and methane metric rather than by a single average percentage.

Propionibacteria illustrate the importance of the basal diet. In beef heifers fed a 70:30 forage-to-concentrate diet, three Propionibacterium strains did not change daily CH_4_ production, although CH_4_ per kilogram DMI was 8% to 13% lower; the authors attributed this intensity response largely to numerically greater intake, and the inoculated bacteria showed limited persistence (Vyas et al. 2014a). In a separate 10:90 forage-to-concentrate finishing diet, the same strain concept did not change either CH_4_ production or yield, likely because the high-starch diet already supported substantial propionate formation (Vyas et al. 2014b). In dairy cows, *Propionibacterium freudenreichii* P63 alone or with lactobacilli produced diet- and strain-dependent responses; only one combination on the low-starch diet tended to lower CH_4_ intensity, by about 26.5% when expressed per kilogram of milk or 4% fat-corrected milk (Philippeau et al. 2017).

Lactic acid bacteria also show strong strain and host dependence. Jeyanathan et al. (2016) selected three bacterial strains after in vitro screening and tested them in sheep*. Lactobacillus pentosus* D31 reduced CH_4_ by about 13%, whereas another selected strain increased CH_4_ by about 16% and the third had no effect. The same general strain-selection approach did not translate to lactating dairy cows: none of three bacterial DFMs reduced CH_4_, and *P. freudenreichii* increased CH_4_ intensity under the high-starch diet in one treatment period (Jeyanathan et al. 2019). More recent cow studies are similarly cautious. A commercial Lactobacillus mixture did not significantly change CH_4_ yield or intensity in 40 lactating cows (Williams et al. 2023), and freeze-dried or liquid lactobacilli did not change CH_4_ production, yield, or intensity in grazing early-lactation cows, despite a numerical 6% reduction in yield with the freeze-dried product (Nair et al. 2026).

Animal data for Bacillus are mixed rather than uniformly negative. In sheep, low and medium doses of *Bacillus licheniformis* reduced daily CH_4_ and CH_4_ per kilogram DMI by about 6% while improving digestibility and energy use (Deng et al. 2018). In 60 lactating Holstein cows, dietary *Bacillus subtilis* (50 g/cow per day) reduced CH_4_ yield by 10% and CH_4_ intensity by 6.8% while increasing DMI and milk yield (Jia et al. 2022). In contrast, a 12-week study with *B. subtilis* 810 and *B. licheniformis* 809 found that dietary starch, not the DFM, reduced CH_4_; the DFM improved nutrient digestibility without a methane effect (Juckem et al. 2025). These studies show why genus-level claims are insufficient.

Conventional live yeast has clearer evidence for rumen modulation than for direct methane suppression. Two active-dried *Saccharomyces cerevisiae* strains did not significantly lower CH_4_ relative to an unsupplemented control in nonlactating cows, although one strain differed from the other and altered rumen fermentation (Chung et al. 2011). In lactating cows, a *S. cerevisiae*-based DFM did not affect CH_4_ production, yield, or intensity but increased milk yield by about 2 kg/d (Oh et al. 2019). This distinction is important: improved production can lower emissions intensity without demonstrating inhibition of methane formation.

Recent microbial strategies expand the evidence base beyond conventional probiotic genera. A rumen-derived methanotroph-methylotroph consortium (NC52PC) reduced daily CH_4_ by 12.0% and 14.4% at low and high doses, respectively, in 12 Hanwoo steers after showing much larger reductions in vitro (Tseten et al. 2025). A native rumen microbial feed supplement containing bacterial and yeast strains improved production outcomes in Jersey cows, but CH_4_ production and yield were unchanged and the reduction in intensity was only a tendency (Valldecabres et al. 2026). Most recently, high-dose *Pichia membranifaciens* M12 reduced daily CH_4_ by 15.4%, CH_4_ yield by 18.7%, and CH_4_ intensity by 17.7% in a 36-cow trial without reducing lactation performance (Li et al. 2026). These emerging results are important, but they remain single-study evidence and require independent replication.

Table 2 presents representative in vivo studies with direct methane measurements. Together, the studies show a consistent pattern: microbial interventions can affect CH_4_, but the direction and magnitude are not predictable from microbial genus alone. The same class can produce a favorable response, no response, or an unfavorable response depending on strain, diet, host, dose, duration, and persistence.

### Function-first framework for DFM development

The animal evidence supports a function-first rather than taxonomy-first development strategy. Candidate DFMs should be selected for a measurable methane-relevant function rather than because they belong to a genus commonly regarded as probiotic. Table 3 integrates proposed functions, representative in vivo responses, evidence readiness, confirming metrics and development priorities. The framework uses three principal readiness tiers: Tier 1, mechanistic plausibility; Tier 2, controlled in vivo efficacy that remains context-dependent or insufficiently replicated; and Tier 3, sustained translational efficacy demonstrated across independent or farm-relevant studies without compromising productivity, safety, or feasibility. Intermediate Tier 1–2 classifications are used where mechanistic support is accompanied by limited animal evidence. No DFM class can yet be considered uniformly Tier 3.

**Table 3:** Function-first decision framework for DFM development and farm-scale methane validation.

Targeted function	Candidate/strategy	**Current** in vivo **signal**	Evidence tier	Key confirming metric	Development decision
**Propionate enhancement/H_2_ redirection**	*Propionibacterium*; selected lactobacilli	Mixed: null daily CH_4_ in several cattle studies; strain/diet-specific effects	Tier 2, inconsistent	CH_4_ yield + VFA profile + strain persistence	Use diet-stratified trials; avoid genus-wide claims.
**Rumen stabilization/O_2_ scavenging**	Saccharomyces cerevisiae	Usually no direct CH_4_ reduction; production responses may occur	Tier 2, indirect	CH_4_ intensity + pH + intake + performance	Position primarily as rumen-efficiency strategy unless direct CH_4_ effect is shown.
**Fermentation/community modulation**	*Bacillus* spp.	Modest reductions in some sheep/cow studies; null responses in others	Tier 2, context-dependent	Daily CH_4_ + yield + diet interaction	Replicate by strain and basal diet before mitigation claim.
**Direct antagonism**	Bacteriocin-producing bacteria	Mostly mechanistic/experimental	Tier 1	CH_4_ + active compound + methanogen response	Continue mechanism, dose, delivery, and safety studies.
**Alternative electron sink**	Reductive acetogens; nitrate-supporting microbes	Limited animal efficacy for acetogens; nitrate-supporting microbes may improve nitrite handling	Tier 1–2	CH_4_ + H_2_/redox balance + safety end points	Require persistence and safety before scale-up.
**Methane consumption**	Methanotroph-methylotroph consortia	12.0%–14.4% lower daily CH_4_ in one steer study	Tier 2, emerging	Daily CH_4_ + methane yield + consortium recovery	Independent replication and longer trials needed.
**Rumen-adapted yeast modulation**	Rumen-derived *Pichia membranifaciens*	High dose reduced production, yield, and intensity in one 36-cow study	Tier 2, emerging	All CH_4_ metrics + productivity + microbiome/function	High priority for independent validation.
**Multifunctional consortia**	Defined compatible strain combinations	Meta-analytic subgroup signal but heterogeneous formulations	Tier 1–2	CH_4_ production, yield, intensity + strain recovery	Design by complementary functions; validate composition and persistence.

Tier 1, mechanistic plausibility; Tier 2, controlled in vivo efficacy that is context-dependent or not yet independently replicated; Tier 3, sustained translational efficacy across farm-relevant settings with acceptable productivity, safety, and feasibility. No DFM class is currently assigned uniformly to Tier 3.

The framework also distinguishes methane outcomes from mechanisms. A reduction in CH_4_ is an outcome, whereas a mechanism requires evidence for the biological process responsible for that response. Such processes may include enhanced propionate formation, redirection of reducing equivalents, methane utilization by an administered consortium, production of antimethanogenic metabolites, or reproducible changes in microbial interactions linked to the observed CH_4_ response. Table 3 therefore links each major DFM strategy with its proposed function, current in vivo signal, evidence readiness, and requirements for further development.

### Mechanisms of DFM action

Redirection of metabolic hydrogen is the most frequently proposed mechanism. Propionate-forming and lactate-utilizing pathways can capture reducing equivalents that might otherwise support hydrogenotrophic methanogenesis. This does not mean that ruminal H_2_ production is eliminated. Rather, the distribution of electron flow among competing fermentation pathways is altered (Janssen 2010; Greening et al. 2019; Ungerfeld 2020). A favorable shift in VFA profile is therefore supportive evidence, but it should be accompanied by a direct CH_4_ measurement before a mitigation claim is made.

Alternative electron sinks provide a related route. Reductive acetogenesis, nitrate reduction, sulfate reduction, and fumarate- or succinate-linked pathways can consume reducing equivalents, but each has ecological or safety constraints. In particular, acetogens usually compete poorly with methanogens in the mature rumen, and nitrate strategies require control of nitrite accumulation (van Lingen et al. 2016; Guyader et al. 2017; Ungerfeld 2020). Microbial products intended to support these pathways should therefore be evaluated with end-product measurements and, where relevant, safety markers rather than CH_4_ alone.

Direct or indirect antagonism of methanogens is another possible mechanism. Bacteriocins and other antimicrobial metabolites can suppress methanogens or organisms that supply methanogenic substrates, but effects must be demonstrated for the specific producer and compound. Evidence from bovicin-producing streptococci illustrates the concept but remains predominantly mechanistic rather than farm-ready (Garsa et al. 2019).

Some DFMs may act primarily by improving rumen stability. Live yeast and selected bacteria can influence oxygen removal, pH, fiber digestion, and microbial interactions (Seo et al. 2010; Jeyanathan et al. 2014). These effects may improve production efficiency without reducing daily CH_4_. Consequently, rumen stabilization should be evaluated together with intake, digestibility, VFA profile, CH_4_ production, CH_4_ yield, CH_4_ intensity, and animal performance.

Persistence is a cross-cutting requirement. An administered microbe may fail because it does not survive feed processing, feeding, or rumen turnover, even when its mechanism is plausible in vitro. Limited persistence was documented for Propionibacterium in beef heifers and was proposed as one reason for weak methane responses (Vyas et al. 2014a, 2014b). Trials should therefore report viable dose at feeding and, when feasible, strain recovery or a validated marker of functional activity. Figure 3 summarizes these linked mechanisms and response modifiers.

**Figure 3:**
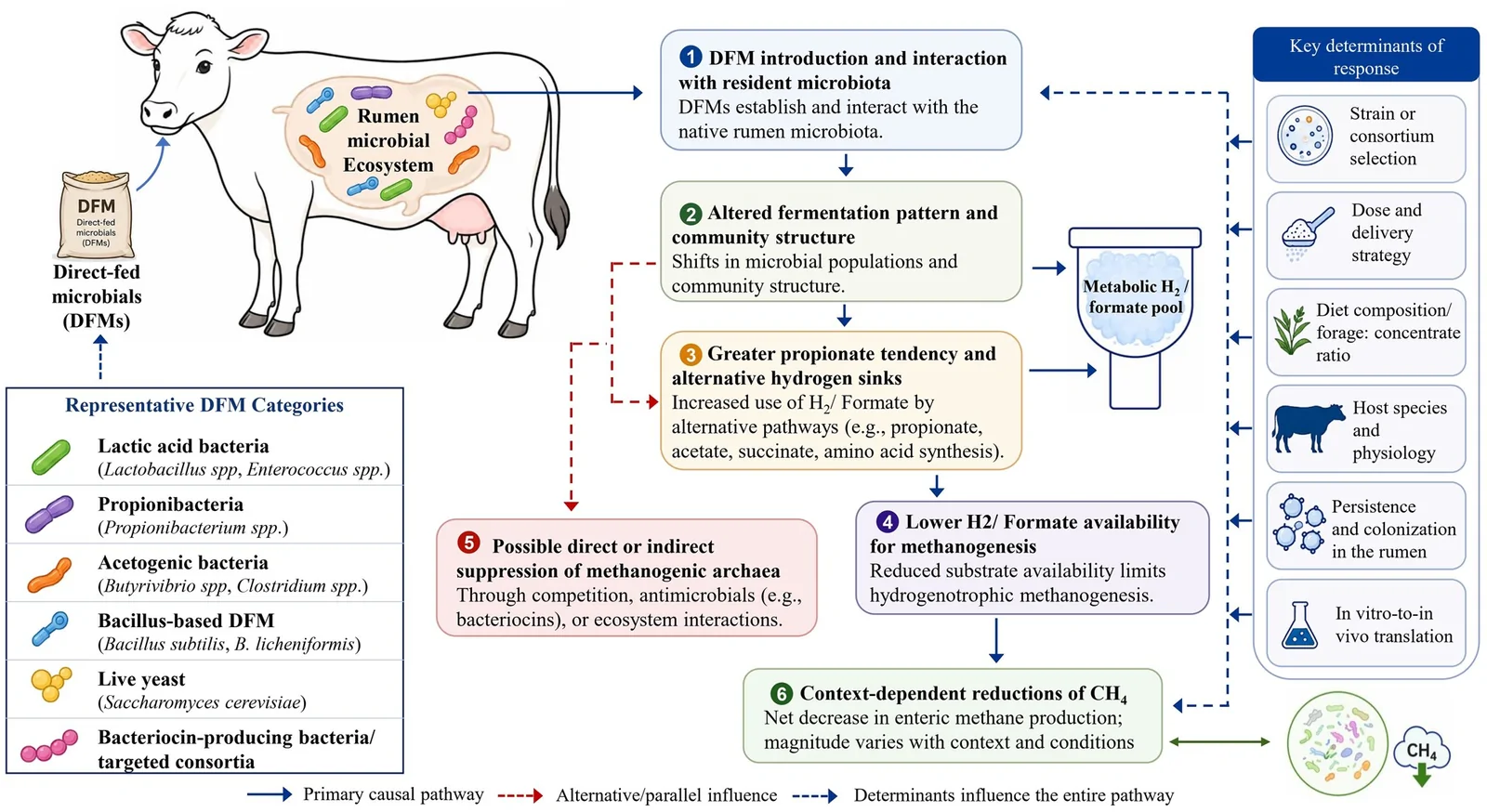
Conceptual mechanisms by which DFMs may alter enteric methane. Administered microbes interact with the resident rumen community and may change fermentation, redirect H_2_/formate and reducing-equivalent flux toward competing pathways, stabilize the rumen environment, or exert direct or indirect effects on methanogenic archaea. The final CH_4_ response depends on strain, dose, delivery, diet, host, persistence, and measurement context (Jeyanathan et al. 2014; Greening et al. 2019; Ncho et al. 2024).

### In vitro-to-in vivo translation

In vitro systems are useful for screening many strains under controlled substrate, inoculum, dose, and incubation conditions. They cannot reproduce feed intake, rumen turnover, absorption, host feedback, long-term adaptation, or competition within the intact rumen. This limitation is visible in the DFM literature: strains selected after favorable in vitro screening have produced smaller, null, or opposite responses when transferred to sheep or cattle (Jeyanathan et al. 2016, 2019). The NC52PC consortium provides a second example; methane suppression exceeded 40% in vitro but was 12.0% to 14.4% in steers (Tseten et al. 2025).

Measurement also affects interpretation. Respiration chambers provide controlled and precise short-term measurements, whereas GreenFeed and SF_6_ methods allow repeated measurements under less restrictive conditions but introduce different sources of variation (Knapp et al. 2014; Patra 2016). Methane method, adaptation period, and number of observation days should therefore be considered part of the evidence context rather than background details.

Farm adoption adds another layer. Product stability, mixing uniformity, storage, repeatable viable dose, compatibility with local diets, regulatory status, safety, cost, and production response can determine whether a statistically detectable effect is useful. Figure 4 summarizes the main barriers between laboratory promise and farm-scale application.

**Figure 4:**
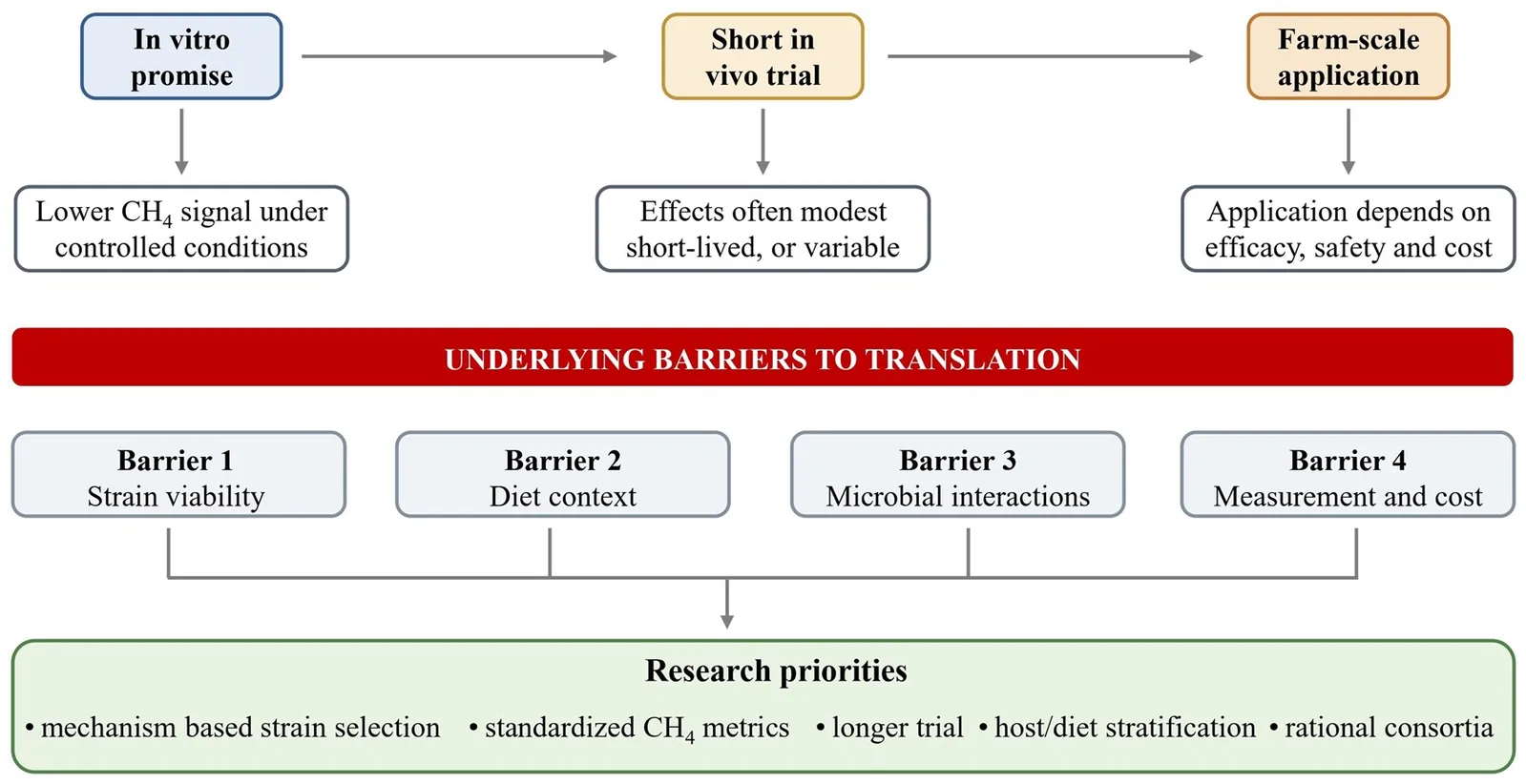
Main barriers to translation of DFM methane responses from controlled experiments to farm use. In vitro screening can identify mechanistic candidates, but animal and farm responses may be modified by strain viability, diet, microbial competition, host physiology, trial duration, methane-measurement method, product stability, and cost (Jeyanathan et al. 2019; Ncho et al. 2024; Tseten et al. 2025).

### Biological relevance of methane responses

Statistical significance and biological importance are not equivalent. A small statistically significant change may have little practical value if it is transient, costly, or accompanied by lower intake or productivity. Conversely, a modest change in CH_4_ intensity may be relevant when it is sustained and results from higher productive output without greater daily CH_4_. The denominator therefore matters (Knapp et al. 2014).

The in vivo DFM literature provides several examples. In the high-forage Propionibacterium study, CH_4_ per kilogram DMI was lower even though daily CH_4_ was unchanged, and the authors related the intensity difference to intake (Vyas et al. 2014a). In the *S. cerevisiae* study of Oh et al. (2019), milk yield increased while CH_4_ production, yield, and intensity were unchanged. In contrast, high-dose *P. membranifaciens* M12 reduced all three-methane metrics without reducing lactation performance (Li et al. 2026). These outcomes have different biological meanings even when each is described as a methane-related response.

For this reason, future DFM studies should report at least daily CH_4_ production, CH_4_ yield per kilogram DMI, and CH_4_ intensity per unit of product, alongside DMI, digestibility, and productive performance. Percentage change should always be linked to an absolute metric and a clearly defined denominator.

### Integration with established methane-mitigation technologies

Microbial additives are unlikely to replace the most effective established methane inhibitors in the near term. A more useful question is whether a DFM can complement an intervention by stabilizing fermentation, redirecting reducing equivalents, or improving safety and productivity. Direct evidence for such combinations is still limited, so this section distinguishes demonstrated combinations from mechanistic hypotheses.

Nitrate provides the clearest proof of concept. Nitrate competes for reducing equivalents but carries a risk of nitrite accumulation. In cannulated Holstein steers, the nitrite-selected bacterium Paenibacillus 79R4 accelerated nitrite handling, prevented methemoglobin accumulation, and reduced adverse fermentation effects when administered with nitrate (Latham et al. 2019). That experiment supports a microbial safety-function approach to nitrate use, although it did not establish a sustained combined methane response over a production cycle.

For 3-nitrooxypropanol (3-NOP), methane inhibition can increase the need to dispose of reducing equivalents through nonmethanogenic routes. The efficacy of 3-NOP is well established and is modified by dose and diet composition (Hristov et al. 2022; Kebreab et al. 2023). A DFM selected to enhance propionate formation or another productive electron sink is therefore mechanistically attractive as a partner, but controlled in vivo evidence for a DFM + 3-NOP combination is presently insufficient. The same caution applies to bromoform-rich seaweed: large methane reductions have been demonstrated with *Asparagopsis taxiformis*, but evidence that a DFM improves the persistence, productivity, or hydrogen balance of this response remains sparse (Roque et al. 2021; Roques et al. 2024). Combination studies should be factorial and should report whether effects are additive, synergistic, neutral, or antagonistic.

### Safety, formulation, and practical application

A methane-oriented DFM must meet the same basic requirements as any microbial feed additive. Candidate strains should be identified to an appropriate taxonomic level and screened for transferable antimicrobial resistance, virulence-associated determinants, toxin production, and other safety concerns relevant to the species used. This is especially important for genera that contain both beneficial and opportunistic strains (Seo et al. 2010; Doyle et al. 2019).

Formulation can determine whether a biologically active strain reaches the animal at the intended dose. Viability may decline during drying, storage, pelleting, transport, or mixing. Dose should therefore be reported as viable cells delivered per animal per day or per unit of feed, with analytical confirmation whenever possible. Multistrain products should report the viable contribution of each component rather than only the total count.

Practical recommendations should also distinguish methane reduction from production benefits. A product can improve fiber digestion, milk yield, or feed efficiency without lowering daily CH_4_, as shown in several yeast and native microbial-product studies (Oh et al. 2019; Valldecabres et al. 2026). Such effects may still be valuable, but the product should not be described as a methane suppressant unless CH_4_ production or yield is directly reduced.

Diet is a primary response modifier. The contrast between the two Propionibacterium beef studies, the diet-dependent response in Philippeau et al. (2017), and the starch-by-DFM study of Juckem et al. (2025) all show that a microbial product should be evaluated in the feeding system for which it is intended. This is especially important in grazing and smallholder systems, where forage quality, concentrate use, and daily delivery differ from controlled total mixed ration systems.

### Research priorities

Future work should begin with a defined function and a defined target system. Candidate strains should be screened for methane-relevant phenotypes such as propionate formation, lactate cross-feeding, alternative electron use, methanotrophy, antimicrobial production, or rumen persistence. Genomic and multi-omics data are useful when they are linked to these measured phenotypes rather than used as stand-alone evidence (Greening et al. 2019; Li et al. 2026).

Animal trials should then test whether the function survives translation. At minimum, studies should report strain identity, viable dose, basal diet, adaptation and supplementation duration, methane method, daily CH_4_, CH_4_ yield, CH_4_ intensity, DMI, rumen fermentation, and animal performance. Longer trials and independent replication are particularly important for the recent methanotroph and rumen-derived yeast approaches (Tseten et al. 2025; Li et al. 2026).

Rational consortia remain a high-priority research direction because different organisms may perform complementary functions. The positive subgroup signal for multistrain bacterial products in the cattle meta-analysis supports this possibility, but it does not establish that any particular consortium will work across diets (Ncho et al. 2024). Compatibility, stability, persistence, and safety should therefore be demonstrated before complex consortia are advanced to large animal trials.

Finally, future studies should compare DFMs alone and in combination with established technologies. Such experiments can test whether microbial functions recover useful electron flow, reduce safety constraints, or maintain productivity when methanogenesis is strongly inhibited. This would move the field from testing isolated products toward designing biologically coherent mitigation packages.

## Conclusions

Direct-fed microbials can influence enteric methane, but the current animal evidence does not support a universal DFM effect. Conventional lactobacilli, propionibacteria, Bacillus, and live yeast have produced mixed responses, including reductions, null effects, and occasional unfavorable changes. Recent methanotroph-based consortia and rumen-derived yeasts are promising, but they remain early evidence rather than established farm technologies.

The most useful development strategy is therefore function-first. A candidate should be linked to a measurable mechanism, tested in the diet and host for which it is intended, and advanced according to evidence readiness. The target is not elimination of normal H_2_ production, but productive redirection of reducing equivalents away from methanogenesis while preserving rumen function.

A farm-relevant methane claim should require persistent improvement in CH_4_ production, yield, or intensity together with acceptable intake, digestibility, health, productivity, safety, and cost. Applying these criteria should make future DFM studies easier to compare and should clarify which microbial strategies are ready for translation and which remain proof of concept.

## Funding

None declared.

## Data Availability

No new experimental data were generated in this narrative review. The literature sources used for the revised evidence synthesis are cited in the manuscript.

## List of abbreviations

3-NOP: 3-nitrooxypropanol
CH_4_: methane
DFM: direct-fed microbial
DMI: dry matter intake
ECM: energy-corrected milk
H_2_: molecular hydrogen
RUSITEC: rumen simulation technique
SF_6_: sulfur hexafluoride
VFA: volatile fatty acid
